# Breeding habitat loss reveals limited foraging flexibility and increases foraging effort in a colonial breeding seabird

**DOI:** 10.1186/s40462-020-00231-9

**Published:** 2020-11-10

**Authors:** Marwa M. Kavelaars, Jan M. Baert, Eric W. M. Stienen, Judy Shamoun-Baranes, Luc Lens, Wendt Müller

**Affiliations:** 1 Behavioural Ecology and Ecophysiology (BECO) Researchgroup, University of Antwerp, Universiteitsplein 1, 2610 Antwerp, Belgium; 2 Terrestrial Ecology Unit (TEREC), Ghent University, K.L. Ledeganckstraat 35, 9000 Ghent, Belgium; 3 Research Institute for Nature and Forest (INBO), Kliniekstraat 25, 1070 Brussels, Belgium; 4 Computational Geo-Ecology, IBED, University of Amsterdam, P.O. Box 94248, 1090 GE Amsterdam, The Netherlands

**Keywords:** GPS tracking, Foraging behaviour, Behavioural plasticity, Habitat destruction, Lesser black-backed gull, Seabirds, Early development

## Abstract

**Background:**

Habitat loss can force animals to relocate to new areas, where they would need to adjust to an unfamiliar resource landscape and find new breeding sites. Relocation may be costly and could compromise reproduction.

**Methods:**

Here, we explored how the Lesser black-backed gull (*Larus fuscus*), a colonial breeding seabird species with a wide ecological niche, responds to the loss of its breeding habitat. We investigated how individuals adjusted their foraging behaviour after relocating to another colony due to breeding site destruction, and whether there were any reproductive consequences in the first years after relocation. To this end, we compared offspring growth between resident individuals and individuals that recently relocated to the same colony due to breeding habitat loss. Using GPS-tracking, we further investigated the foraging behaviour of resident individuals in both colonies, as well as that of relocated individuals, as enhanced foraging effort could represent a potential driver of reproductive costs.

**Results:**

We found negative consequences of relocation for offspring development, which were apparent when brood demand was experimentally increased. Recently relocated gulls travelled further distances for foraging than residents, as they often visited more distant foraging sites used by residents breeding in their natal colony as well as new areas outside the home range of the residents in the colony where they settled.

**Conclusions:**

Our results imply that relocated individuals did not yet optimally adapt to the new food landscape, which was unexpected, given the social information on foraging locations that may have been available from resident neighbours in their new breeding colony. Even though the short-term reproductive costs were comparatively low, we show that generalist species, such as the Lesser black-backed gull, may be more vulnerable to habitat loss than expected. Long term studies are needed to investigate how long individuals are affected by their relocation in order to better assess potential population effects of (breeding) habitat loss.

## Introduction

Abrupt changes in habitat availability due to human activities may pose major challenges for species that rely on spatial knowledge for finding their food sources, in particular for central place foragers during the reproductive period [1–3]. Searching for new food sources can require a substantial amount of time and energy [4], which reduces foraging efficiency, and may ultimately affect fitness [5]. It is generally assumed that the dynamics of populations under such rapid and unpredictable environmental changes are affected by the ease with which individuals adapt to unfamiliar food sources. How individuals adapt, in turn, depends on the degree of individual plasticity as well as the width of their ecological niche, which is determined in part by their individual foraging specialisation [6–8].

Individual foraging specialisation is common in many species [6, 9], with individuals varying in resource selection (i.e. the type of food eaten, the variety of the diet) or foraging site fidelity, both may require specific foraging skills, experience, social status or prior knowledge [10–13]. Specialised foraging behaviour should be favoured when it improves foraging efficiency, by reducing the time or energy required to search or capture food [14, 15]. This is particularly beneficial when a considerable time and energy investment is required to learn the skills to successfully capture particular prey items [16–18]. Specialisation may, for example, improve foraging success through prior knowledge of food sources when they are scarcely distributed [19–21]. Specialists benefit especially when resources are predictable in time and space [22], as their (local) knowledge only increases foraging efficiency if that information is reliable [9, 23]. Furthermore, specialists can avoid competition by specialising on only a small range of the ecological niche [6].

However, restricted use of foraging techniques, sites and resources might at the same time render specialists vulnerable to unstable environmental conditions [24]. For example, when animals are forced to leave their original area and settle in a new one (from here on ‘relocation’) due to habitat loss or other environmental changes, their specialised foraging skills or former spatio-temporal knowledge may be less beneficial [3]. Therefore, specialists are generally expected to endure a greater impact of environmental fluctuations than generalists that can make use of a variety of different resources [1, 2].

The extent to which specialists can adjust their foraging strategies affects their vulnerability to environmental changes. This may depend on whether foraging specialisation relates to morphological or behavioural skills, or whether it relies on spatio-temporal knowledge [6, 9]. Morphological plasticity is often limited while behavioural traits are assumed to be more flexible [24, 25]. Spatial knowledge must be gained anew when encountering a novel environment. Finally, the adjustment to new environments could be facilitated in species that forage in flocks or breed in colonies where learning from conspecifics may allow them to gain information about foraging opportunities [26–29]. However, as of yet, studying how individuals adjust to new environments, and addressing potential costs of changes in foraging behaviour, has largely been hampered by the difficulties of following individuals from free-ranging populations for extended periods of time.

In this study, we investigate the consequences of relocating from a breeding site which was partially destroyed to an alternative breeding site in Lesser black-backed gulls (*Larus fuscus*). Lesser black-backed gulls are considered to be a generalist species that exploits a wide range of natural and anthropogenic food sources at the species level [30], although individuals may vary in diet choice (i.e. terrestrial, urban, or marine food), spatio-temporal consistency in foraging behaviour, as well as in the level of specialisation [31–34]. Following the loss of substantial parts of breeding habitat in the port of Zeebrugge, Belgium, due to the 2014-expansion of warehouses and fox predation, the population declined by 75% and a large number of gulls left the colony in search of alternative breeding sites [35]. Many of these colour-ringed birds could be traced back as they subsequently settled in a nearby (37 km) colony in the industrial port of Vlissingen, the Netherlands, which is within the daily foraging range of Lesser black-backed gulls. To assess the consequences of this relocation on foraging efficiency and reproduction, we tested if offspring development in the Vlissingen colony varied depending on the origin of the parents by comparing pairs with at least one relocated parent with resident pairs. In all nests, offspring demand was experimentally altered via brood size manipulations, as effects may especially emerge when conditions to raise chicks are more challenging. To test the hypothesis that adverse effects on growth after relocation mainly result from unfamiliarity with new environments, we used GPS-tracking devices to compare foraging behaviour between relocated individuals, resident individuals in Vlissingen (new colony), and resident individuals still breeding in remaining parts of the colony in Zeebrugge (old colony).

## Material and methods

### Field sites and identification of birds

In 2015–2017, fieldwork was carried out from mid-April until mid-July in the colonies of Vlissingen, the Netherlands (51° 27′ N, 3° 42′ E), and Zeebrugge, Belgium (51° 20′ N 3° 10′ E) (respectively approximately 4500 and 1500 ground-breeding pairs), which lie 37 km apart from each other. We searched both colonies for Lesser black-backed gulls (*Larus fuscus*) of known origin based on the presence of colour and metal rings and re-sightings in either of the two colonies during previous breeding seasons. This allowed us to assign individuals to one of three groups: resident individuals in the old Zeebrugge colony (zb zb), resident individuals in the Vlissingen colony (vl vl), and individuals that relocated from Zeebrugge to Vlissingen (zb vl). Individuals breeding in their natal colony were categorised as ‘resident’. In Vlissingen we found many gulls with a Zeebrugge origin, but not vice versa, supporting our assumption that the unidirectional relocation was triggered by the substantial destruction of breeding habitat in Zeebrugge, which took place in 2014. Individuals in Vlissingen that were originally colour-ringed in Zeebrugge and were known to breed in their natal colony prior to relocating (2013–2014), were categorised as ‘relocated’ and followed during their first breeding attempt in their new colony. The study colonies were visited every 2–3 days from the onset of egg laying, and the laying date of each egg was indicated using a non-toxic marker, which allowed us to identify laying order.

### Effects of relocation on chick development

To assess the effect of relocation on offspring development, an experiment was performed in the new colony Vlissingen. Pairs with at least one relocated parent (zb vl) and resident pairs (vl vl) were randomly divided into nests with low demand (1 chick, resident: *n =* 31 nests, relocated: *n =* 21 nests) and high demand (3 chicks, resident: *n =* 37 nests, relocated: *n =* 18). Lesser black-backed gulls lay three-egg clutches and hatch their eggs asynchronously. In our study colonies they typically raise 1–2 chicks per year (E. Stienen and R.J. Buijs pers. communication). For 23 of the pairs that were followed over multiple years, the brood size manipulation was alternated between years. At hatching, the complete clutch of the focal pairs was replaced by one (low demand) or three (high demand) unrelated pipping eggs that were randomly taken from surrounding donor nests. Based on the laying date marked on each egg, we only selected first or second laid eggs from donor nests, as the third egg is often of lower quality [36]. This way, we avoided the occurrence of any runt chicks in the nests, as hatching order related mortality is common [37, 38], and ensured that all eggs hatched synchronously. This increased the chance of survival of all three chicks in the enlarged nests, thereby maximising brood demand. The eggs of the experimental nests were placed in the nests of the donor pairs or in nests with similar laying dates that were partly depredated and no longer followed. Within 2 days from hatching, chicks were individually marked with coloured tape. Throughout the experiment, chicks were kept in wire enclosures that were put up around each nest prior to hatching (circa 2 × 2 m in size, and 0.3 m high) to ensure that they stayed close to their nest for the entire developmental period. PVC tubes were added to each enclosure to provide shelter for the chicks. Chick development (body mass) was measured every 2–3 days until fledging (day 30) and chick mortality was recorded during each visit. Sex was determined molecularly using down feathers [39].

### Effects of relocation on foraging behaviour

To assess effects of relocation on foraging behaviour, 45 colour-ringed adult Lesser black-backed gulls not included in the brood size experiment were caught for GPS-tracking in Vlissingen and Zeebrugge between mid-May and the beginning of June in three consecutive years (2015–2017). In Vlissingen, we tagged resident individuals (*n =* 8; 2 females and 6 males) as well as relocated individuals from Zeebrugge during their first breeding attempt in Vlissingen (n *=* 8; 1 female and 7 males). In Zeebrugge, we tagged resident individuals (*n* = 29; 15 females and 14 males) breeding in the small remaining part of the colony from which the relocated individuals originated, an area that was undisturbed and fenced off to protect it from fox predation. All birds were caught on the nest with cages in the second or third week of incubation. Standard morphometric measurements were taken before equipping the birds with UvA-BiTS tracking devices via a Teflon wing harness, weighing combined approximately 2.3% of the bird’s body mass (61 × 25 × 10 mm, 13.5 g + 5 g harness; for more detailed information on the UvA-BiTS GPS devices see [40]; for wing harness attachment see [41]). No deleterious effects on behaviour have been found for the attachment of the GPS devices on the gulls in these colonies [42]. GPS fixes were taken every 3 min throughout the breeding season until the end of July.

In order to standardise brood size and offspring demand among broods, the complete clutch of the GPS birds was replaced by two unrelated pipping eggs at the moment of hatching. Nests were monitored every 2–3 days and chick mortality was recorded during each visit.

### Data analyses

All statistical analyses were performed in R [43]. Non-linear mixed effects models were fitted using the nlme package [44], and for the linear mixed effect models the lmer package [45] was used. We report full models following [46]. Normality, collinearity of explanatory variables, homoscedasticity and independence of model residuals were graphically inspected.

#### Offspring development

First, logistic growth curves were fitted for each chick using least square estimation (nls function):

$$ {W}_t=\frac{A}{1+{e}^{K\left(I-t\right)}} $$

in which W_t_ is the body mass (g) at day t, t is the chick age (days), *A* is the asymptotic mass (g), *K* is the growth rate constant (days^− 1^), and *I* is the inflection point of the growth curve (days) [47]. Chicks that died before 30 days old were excluded as their growth curves could not be fitted (excluded chicks in 3-chick nests: resident *n =* 26 out of 111, relocated *n =* 15 out of 54; no chicks were excluded in 1-chick nests).

Next, we tested if offspring growth depended on parental origin or brood size. To this end, growth parameters *A*, *I*, *K* were modelled as a function of chick sex, brood size (1 or 3) and parental origin (relocated or resident in Vlissingen) using linear mixed effects models. All three-way and pair-wise interactions were also included as fixed effects. Year, parent ID, nest ID and chick ID were included as independent random effects to account for dependence in the data.

#### Adult body condition

In order to exclude an effect of parental quality on offspring development, we compared adult body mass between the three GPS-tracked groups: resident Zeebrugge birds, resident Vlissingen birds and relocated Zeebrugge birds. To evaluate the effect of parental origin on adult body mass, we used a linear model with parental origin (zb zb, vl vl, zb vl) as a fixed effect. Due to the low sample size of tracked females, we only tested the effect of parental origin in males. Tukey HSD post hoc tests were carried out for pair-wise differences.

#### Foraging behaviour

To assess if foraging behaviour varied between relocated and resident birds, we first split the GPS data of each individual into separate foraging trips that started with the last GPS fix inside the colony boundaries and ended with the first GPS fix within the colony boundaries. A spatial polygon was created using the colony boundaries, which are clearly visible as the grass plot used for breeding is bordered by factories, waterways and roads. Trips shorter than 30 min and less than 1 km in distance were excluded, as it is likely that the gulls did not forage on these short nearby trips due to very limited resources in the area. For each individual, we calculated (1) the distance to the furthest point per day (maximum distance); (2) cumulative point to point distance, or in other words total daily distance covered (total distance); (3) daily time away from the colony (duration). Next, we tested if these daily foraging parameters differed between resident Zeebrugge birds (zb zb), resident Vlissingen birds (vl vl) and relocated Zeebrugge birds in Vlissingen (zb vl), using linear mixed effect models including parental origin and chick age (0–30 days) and the interaction between parental origin and chick age as fixed effects. Bird ID was included as a random effect to correct for dependence between trips of the same bird. Additionally, to better comprehend the use of the foraging areas, we divided the total trip distance by the maximum distance (straightness of path) fitting linear mixed effect models including parental origin as fixed effect and bird ID as random effect. Furthermore, the R package rptR [48] was used to test the repeatability of the daily foraging parameters described above (maximum distance, total distance and duration) for each individual throughout the tracking period. Repeatability was calculated separately for the three groups (zb zb, vl vl, zb vl).

We used home range overlap to assess to what extent relocated birds used similar foraging areas as resident birds in Vlissingen and Zeebrugge. Home range overlap between individuals was estimated using the bias-corrected Bhattacharyya coefficient for the autocorrelated-Kernel density estimated (AKDE) home ranges, as developed by [49], using the ‘ctmm’ package [50]. Autocorrelated-Kernel density estimation of home ranges were based on an Ornstein-Uhlenbeck process to account for positional and velocity autocorrelation within a finite home range [50]. Confidence intervals of the overlap between the home ranges were used to quantify the strength of spatial interactions between individuals [51], and were used to present a spatial network diagram.

## Results

### Offspring development

**Fig. 1:**
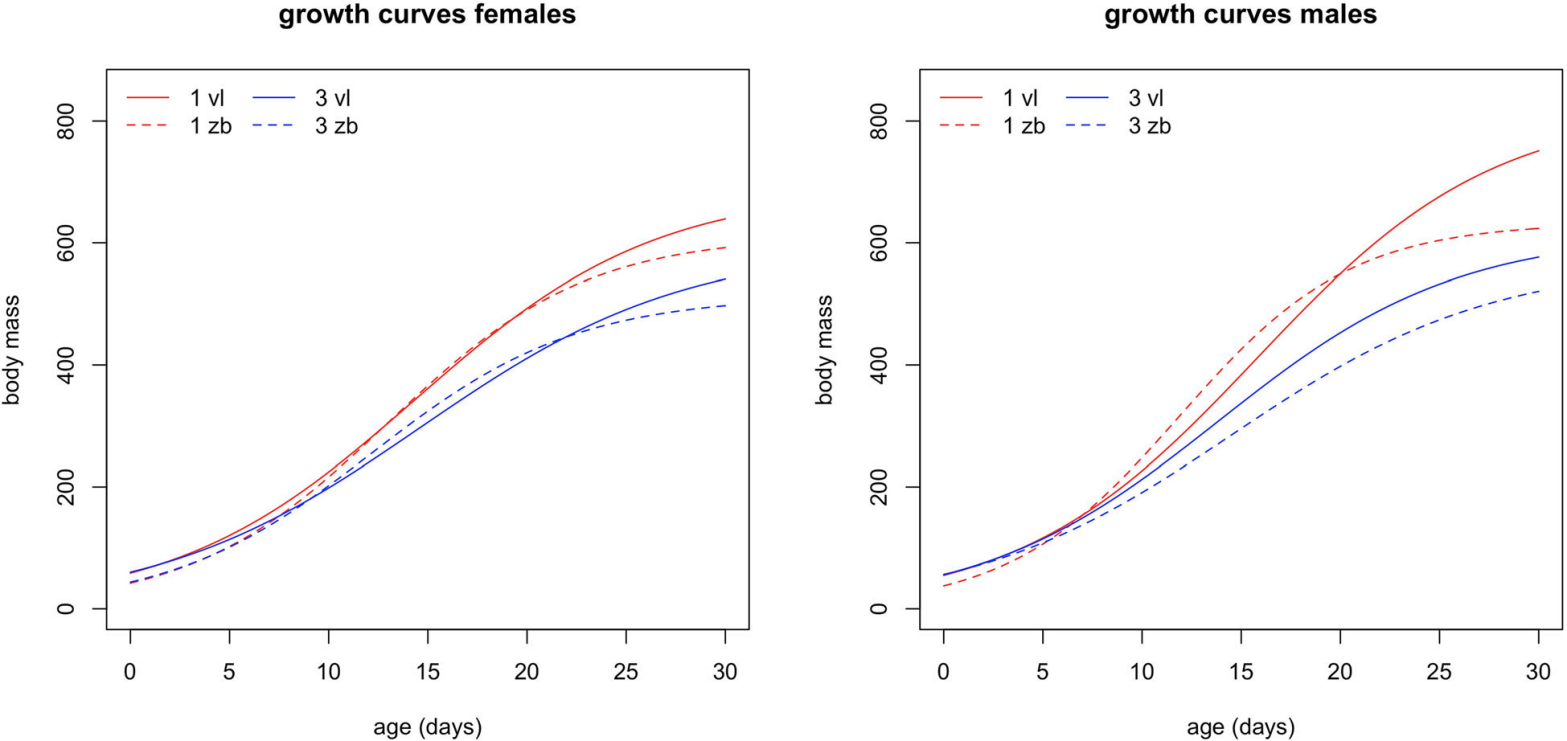
Growth curves based on model estimates for offspring in nests with 1 chick (red) and nests with 3 chicks (blue) for females and males separately. Dashed lines represent chicks of pairs with a relocated parent (zb), solid lines represent offspring of resident pairs (vl) in the new colony (Vlissingen)

The interaction between parental origin and brood size had a significant effect on both the inflection point (*I*) and the growth rate (*K*). Chicks in high demand nests that were reared by pairs with at least one relocated parent had a lower growth rate, and attained half of their fledging weight slower than chicks raised by resident Vlissingen parents did (Fig. 1). However, the growth rate (*K*) did not significantly differ in chicks from relocated and resident parents for single chick nests. In addition, male chicks grew faster than females, independent of parental origin or brood size (Table 1). The asymptotic body mass (*A*), in contrast, only depended on the chick’s sex and brood size (Table 1).

**Table 1:** Full model outcomes of non-linear mixed effects models testing the effects of brood size (1, 3 chicks), chick sex (female, male), parental origin (zb vl, vl vl), the two-way interactions brood size x parental origin, brood size x chick sex, and chick sex x parental origin, and three-way interaction brood size x chick sex x parental origin on three growth parameters: A (asymptotic mass), I (inflection point) and K (growth rate)

Growth parameter	Source of variation	F	d.f.	p
*A*	brood size	164.975	1758	< 0.0001*
*A*	chick sex	55.624	1758	< 0.0001*
*A*	parental origin	17.134	1758	0.9393
*A*	brood size x parental origin	0.006	1758	0.8843
*A*	brood size x chick sex	0.021	1758	0.974
*A*	chick sex x parental origin	0.001	1758	0.36
*A*	brood size x chick sex x parental origin	0.838	1758	0.6339
*I*	brood size	9.841	1758	0.0017*
*I*	chick sex	0.118	1758	0.7313
*I*	parental origin	7.061	1758	0.0079*
*I*	brood size x parental origin	5.21	1758	0.0226*
*I*	brood size x chick sex	0.327	1758	0.5676
*I*	chick sex x parental origin	1.067	1758	0.3018
*I*	brood size x chick sex x parental origin	1.08	1758	0.2989
*K*	brood size	2.067	1758	0.1507
*K*	chick sex	7.988	1758	0.0048*
*K*	parental origin	2.371	1758	0.1238
*K*	brood size x parental origin	4.305	1758	0.0382*
*K*	brood size x chick sex	0.008	1758	0.9274
*K*	chick sex x parental origin	0	1758	0.9907
*K*	brood size x chick sex x parental origin	0.048	1758	0.8265

*Statistically significant

### Adult body condition

Male adult body mass was significantly higher in Zeebrugge residents (947 ± 17 g) than in Vlissingen residents (803 ± 25 g), but there was no significant difference in body mass between relocated birds (854 ± 28 g) and both resident groups (post-hoc Tukey pairwise test: zb zb – vl vl: t = − 4.747, *p* < 0.001; zb zb – zb vl: t = − 2.885, *p* = 0.066; vl vl – zb vl: t = − 1.343, *p* = 0.769).

### Foraging behaviour

**Fig. 2:**
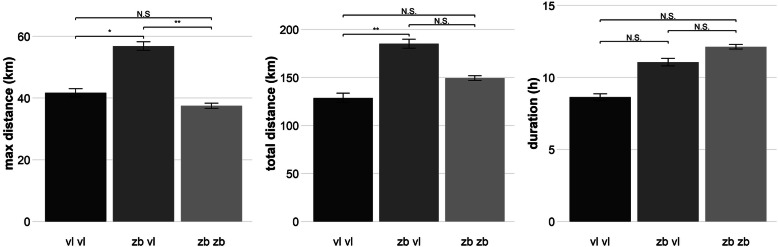
The distance to the furthest point per day (maximum distance (km)), total daily distance covered (total distance (km)) and daily time away from the colony (duration (h)) for the relocated birds (zb vl), the resident birds in the new population (vl vl) and the resident birds in the old population (zb zb). Error bars represent the standard error. Significance codes: < 0.001 ‘***’ 0.001 ‘**’ 0.01 ‘*’

Foraging behaviour of relocated birds differed in several aspects from resident birds in Vlissingen and Zeebrugge (Fig. 2, Tables 2 and 3). On average, the maximum distance that relocated birds travelled was higher than in both resident Vlissingen and Zeebrugge birds (zb zb: 37.0 ± 2.5 km, vl vl: 41.1 ± 4.7 km, zb vl: 54.9 ± 4.7 km; post-hoc Tukey pairwise test: zb zb – vl vl: t = 0.786, *p* = 0.436, zb zb – zb vl: t = 3.402, *p* = 0.001, vl vl – zb vl: t = − 2.082, *p* = 0.043). Additionally, the total distance covered per day was higher in relocated individuals than in resident groups (zb zb: 148 ± 6.82 km, vl vl: 127 ± 13.2 km, zb vl: 180 ± 13.15 km, post-hock Tukey pairwise test: zb zb – vl vl: t = 7.915, *p* = 0.145, zb zb – zb vl: 2.153, *p* = 0.037, vl vl – zb vl: t = − 2.894, *p* = 0.006). The straightness of path differed between the resident groups of both colonies, but both groups did not significantly differ with relocated birds (zb zb: 2.23 ± 0.04, vl vl: 1.89 ± 0.08, zb vl: 2.02 ± 0.08; ﻿post-hoc Tukey pairwise test: zb zb – vl vl: z = − 3.701, *p* < 0.001; zb zb – zb vl: z = − 2.279, *p* = 0.058; vl vl – zb vl: z = − 1.156, *p* = 0.480). The total amount of time away from the colony per day was not significantly different between the three groups (Table 2). Relocated individuals were more repeatable in duration away from the nest than both resident groups, but less in the maximum and total distance travelled (Table 3).

**Table 2:** Full model outcomes of linear mixed effects models estimating the effects of parental origin, chick age and the interaction between parental origin and chick age on the maximum distance per day, total daily distance and daily duration away from colony

	F	df	p
**Maximum distance (km)**			
Parental origin	8.451	60.90	< 0.001*
Chick age	20.012	1192.70	< 0.001*
Parental origin x chick age	2.894	1191.80	0.056
**Total distance (km)**			
Parental origin	6.110	67.17	0.004*
Chick age	47.663	1197.03	< 0.001*
Parental origin x chick age	4.075	1196.11	0.017*
**Duration (h)**			
Parental origin	1.844	64.44	0.166
Chick age	46.580	1195.37	< 0.001*
Parental origin x chick age	13.305	1194.48	< 0.001*

Parental origin is divided in relocated birds (zb vl), resident birds in new colony (vl vl) and resident birds in old colony (zb zb). All models included bird ID as random effect. *Statistically significant *p* < 0.05

**Table 3:** Repeatability of foraging behaviour (maximum distance travelled (km), total daily distance travelled (km), duration away from colony (h)) in relocated Zeebrugge gulls in Vlissingen (zb vl), resident gulls in new colony Vlissingen (vl vl) and resident gulls in old colony Zeebrugge (zb zb)

Foraging behaviour	Group	Repeatability	SE	***p***-value
Maximum distance (km)	zb vl	0.218	0.105	< 0.001
	vl vl	0.327	0.123	< 0.001
	zb zb	0.356	0.066	< 0.001
Total distance (km)	zb vl	0.219	0.102	< 0.001
	vl vl	0.310	0.122	< 0.001
	zb zb	0.272	0.058	< 0.001
Duration (h)	zb vl	0.333	0.124	< 0.001
	vl vl	0.106	0.067	0.001
	zb zb	0.271	0.060	< 0.001

**Fig. 3:**
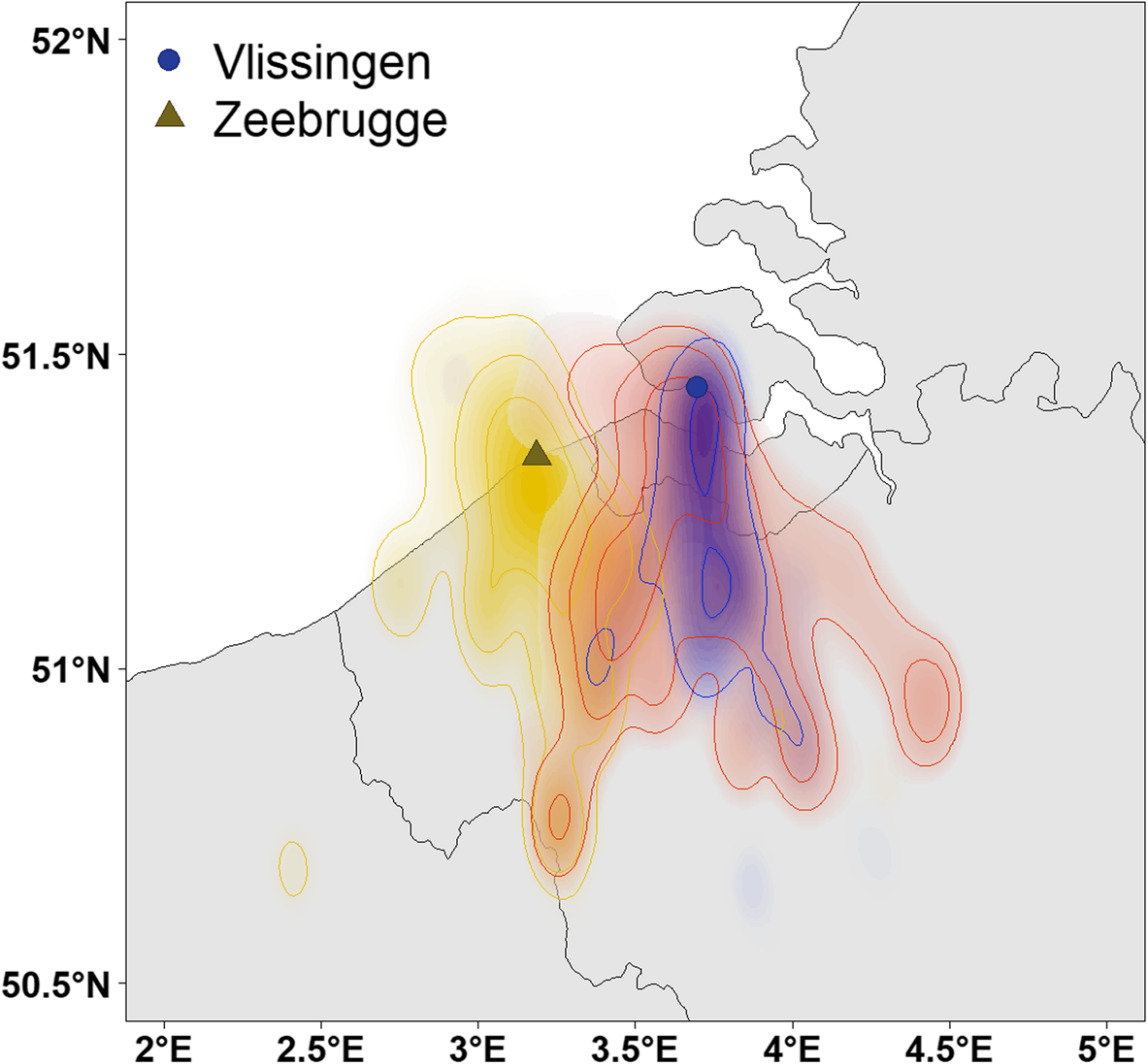
Autocorrelated kernel density distribution of the GPS-tracked Lesser black-backed gulls in Vlissingen (blue dot): relocated birds (vl zb, red, *n* = 8), resident birds of new population (vl vl, blue, n = 8); in Zeebrugge (yellow triangle): resident birds of old population (yellow, *n* = 29). Isopleths represent 25, 50, 75, and 95% of space use by the gulls

**Fig. 4:**
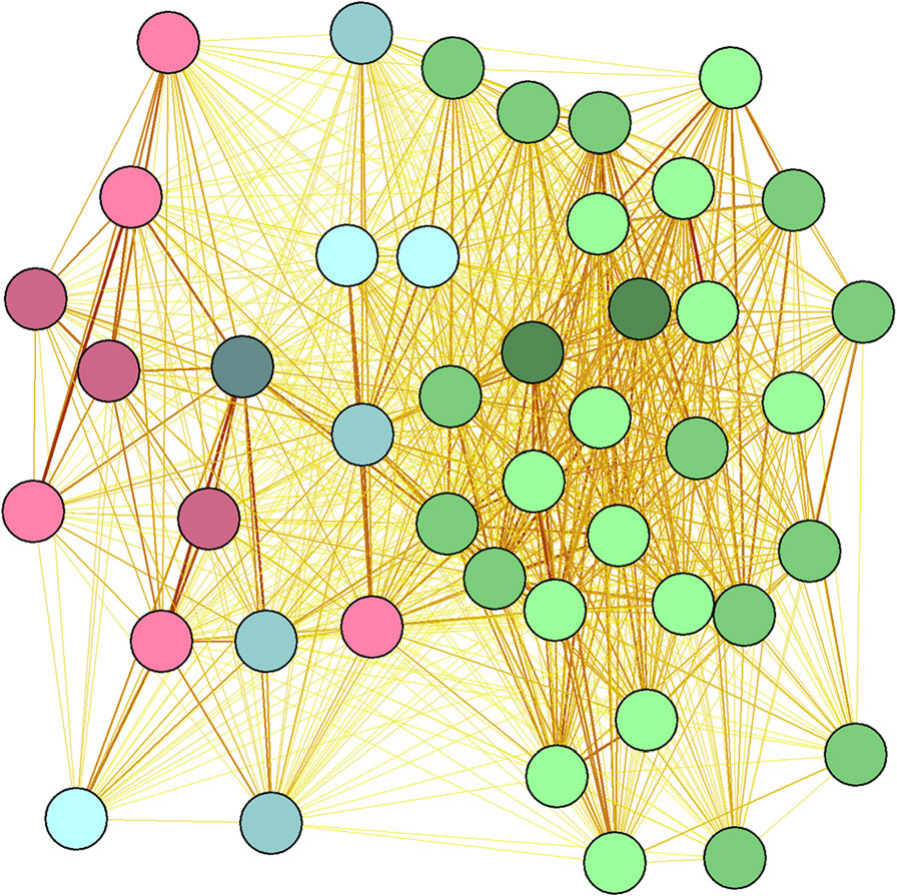
Spatial network diagram based on the estimated home range overlap between individuals for relocated birds (vl zb, blue), resident birds in new population (vl vl, pink), resident birds in old population (zb zb, green). Light tints represent individuals in 2015, intermediate tints represent individuals in 2016, and dark tints represent individuals in 2017. Line colour intensity represents the strength of home range overlap, proximity represents the degree of home range overlap

Home ranges of relocated Zeebrugge birds only partly overlapped with those of resident Vlissingen birds. Relocated Zeebrugge birds still shared part of their home range with resident Zeebrugge birds, and some individuals also visited other areas that were not visited by the resident Vlissingen birds (Fig. 3). This is supported by clustering of home ranges of the GPS-tagged birds in a spatial network diagram based on the strength of home range overlap, demonstrating a strong clustering of resident Zeebrugge and Vlissingen birds with relocated birds taking up an intermediate position (Fig. 4).

## Discussion

Habitat destruction during the reproductive phase in an animal’s annual cycle may force individuals to adjust their foraging behaviour, with potential consequences for their reproductive output. Here, we tested the consequences of a short distance breeding site relocation (37 km) driven by partial habitat destruction on breeding performance and foraging behaviour in the Lesser black-backed gull. Relocated individuals did not use the same foraging areas as their resident neighbours, but instead visited more distant foraging sites, similar to the foraging sites used by individuals from their old colony. Overall, this caused relocated individuals to travel longer distances for foraging than resident birds in the new colony. Growth rates of chicks of relocated birds were significantly lower than those of resident birds when raising a brood of three chicks, and these adverse effects may stem from the unfamiliarity of their parents with the new environment and relatively higher foraging effort. These results were unexpected as our study species is considered to be a generalist seabird species, with a wide ecological niche.

Unforeseen breeding habitat loss in our long-term study colony [35] hence yielded an interesting case study to investigate whether the ability of a species to use a wide niche-range buffers impacts of rapid or unpredictable environmental changes as has been hypothesised [7, 8, 52, 53]. Yet, even though Lesser black-backed gulls tend to exploit a variety of marine and terrestrial food sources (e.g. [54]), relocated gulls seemingly failed to optimally adapt to their new environment, not using foraging sites nearer to their new colony like their resident neighbours. Furthermore, GPS-tracking revealed that relocated gulls still shared a considerable part of their foraging range with birds that were breeding in the remaining part of the colony of Zeebrugge. Note, however, that the sample size is relatively small. The spatial overlap of their foraging area with that of birds of their original colony suggests that most relocated gulls continued to visit familiar foraging sites, causing them to travel longer distances for foraging compared to resident birds in both Zeebrugge and Vlissingen. Such breeding- or foraging site fidelity is common, even during unfavourable conditions [55–57], which suggests that the costs of relocating or changing foraging location may be high. The costs of learning new strategies later in life may be comparable to that of juveniles [58], while competitive exclusion may additionally prevent the access to similar quality foraging sites closer by [6, 59]. Indeed, although the colonies in our study are situated in close proximity of each other, the foraging ranges of the two populations generally do not overlap. This segregation of home ranges of neighbouring colonies is also found in other species and may derive from competition [60–63], which could also explain why relocated individuals did not frequently visit the same areas as their resident neighbours.

Relocated individuals showed a high repeatability in their foraging duration, but not in the distance they travelled. Compared to relocated individuals, both resident groups were more consistent in their foraging distance, but less consistent in the duration away from the nest. The lower repeatability in maximum and total distance travelled, suggests that exploration of the new food landscape was still ongoing in relocated individuals, whereas the residents may know where to go and frequently visit the same areas, resulting in a higher repeatability of foraging distances. Relocated individuals travelled further, but did not spend more time away from the nest, suggesting that there might be a time constraint for the birds when foraging during the chick period [64], as staying away from the colony for too long could increase predation risks for the chicks, especially when the partner leaves as well [65, 66]. As a consequence, relocated birds had less time available for foraging as they spend more time commuting.

Our brood manipulation experiment showed that the extra distance may have hindered relocated birds in a later reproductive stage, as reflected in reduced offspring growth when accommodating the high food demand of three chicks. However, we may underestimate the effect of relocation e.g. if a relocated individual was paired up with a resident, who may partially compensate for a lower provisioning rate by the relocated parent. Our GPS-tracking data suggests that the effect is partly driven by reduced foraging efficiency as a result of unfamiliarity with the new environment, but reduced offspring growth could also relate to a number of additional factors. For example, they may be related to inherent quality differences between relocated and resident birds [67]. Yet, a comparison of the adult body masses implied that residents were not of different quality than relocated birds. Unfortunately, there is no data available on reproductive success of relocated individuals prior to relocation, which could have helped us to investigate changes in parental investment at the individual level. The longer commutes to known foraging sites caused by the relocation may result in higher energetic foraging costs [68], even though this might still be less costly when compared to foraging in a novel yet unexplored environment [69]. Our study reveals that the relocated individuals share some of these costs with their offspring, as exposed by our brood size manipulation experiment. That costs are transferred to the offspring is common in long-lived species [70, 71]. Parents may even cease their current breeding attempt if that would benefit them on the long term [72, 73].

The apparent return to familiar foraging habitats after relocation is remarkable. But evidence has been mounting that many animals, including our study species, display a high level of individual foraging specialisation and site fidelity [20, 21, 34]. Such individual foraging strategies likely involve strong spatio-temporal knowledge [74, 75], and individuals have to familiarise themselves with foraging locations or temporal patterns of food availability [76, 77]. Especially in long-lived species, it may be more efficient to exploit food resources at known locations, instead of spending time on finding new ones [78]. In our study, information on resource availability must have been available for relocated individuals as they were surrounded by residents, so they could potentially use social information [26, 79]. However, information transfer and social learning could be a slow process [29], even within the dense aggregation of a breeding colony.

Finally, while we studied a generalist species with a wide ecological niche, the response to and consequences of (breeding) habitat loss were mainly driven by spatial consistency in foraging behaviour within individuals. This spatial knowledge might differ between the two colonies as well as between individuals [80, 81]. Thus, ultimately, we need to know how foraging specialisation predisposes individuals towards environmental change. Such individual-based approaches gained substantial attention in ecological and behavioural ecological studies [82, 83], but this is given our sample size beyond the possibilities of our study. Yet, individual variation in behaviour may also be important for the interpretation of our study in a different context. We focused on relocated individuals and, as such, we have a non-random sample of individuals that chose to alter their breeding site. Behavioural traits, such as boldness, may have influenced the likelihood of an individual to acquire a new territory or the likelihood to disperse [80, 84, 85]. Shy individuals, for example, could be more likely to become ‘floaters’ that familiarise themselves with new foraging areas before starting to breed, rather than first settling in a new breeding colony and then exploring nearby foraging areas [86]. So, these birds might be underrepresented in our sample of relocated birds. Shy individuals might also be more likely to stay close to their old nesting site, queuing to takeover a nesting spot in the remaining part of their natal colony [87, 88]. This would delay the next reproductive event, but the costs of foraging may remain constant. However, recently it was shown for this population that even a small-scale relocation within the colony due to local breeding area loss can lead to a decrease of clutch volume, i.e. reduced parental investment [89], indicating the complexity of capturing the costs and benefits of behavioural strategies when the environment changes. It nevertheless underlines how individual variation in behavioural traits influences the response to environmental change and hence population-level processes [90]. This might alter the diversity of foraging strategies or animal personalities in a given population, which will impinge on the resilience of a population to subsequent environmental disturbances. This once more highlights the need for individual-based approaches to understand population dynamics in in the context of global change.

## Conclusions

Nowadays, many animal species inhabit anthropogenic landscapes, where they witness unparalleled changes that expose them to novel conditions at a rate that they have not experienced over the course of their evolutionary history. These disturbances may overrule the adaptive significance of behavioural strategies. This may ultimately result in stress and reduced fitness, which we, here, illustrate for one of these behavioural strategies, namely individual foraging specialisations. Such individual strategies are thought to represent central mechanisms by which individuals can increase their foraging efficiency. They require experience and spatio-temporal knowledge of reliable food sources, but this knowledge can potentially not be updated at a pace that may be required in these circumstances. The negative effects on reproduction revealed hidden costs of relocation, which are, as yet, often neglected or difficult to assess. At the population level, such effects could alter the distribution of specialists and generalists and thus niche variation. Studying the links between (behavioural) ecological and evolutionary processes will hence become increasingly relevant to understand how animals respond to anthropogenic changes in their environment.

## Data Availability

The datasets supporting the conclusions of this article are available on 10.5281/zenodo.4244454.
